# Concomitant arrhythmia surgery should be a standard procedure in AF patients undergoing cardiac surgery

**DOI:** 10.1111/jocs.16778

**Published:** 2022-07-23

**Authors:** Claudia A. J. van der Heijden, Elham Bidar, Bart Maesen

**Affiliations:** ^1^ Department of Cardiothoracic Surgery Maastricht University Medical Centre Maastricht The Netherlands; ^2^ Cardiovascular Research Institute Maastricht Maastricht University Maastricht The Netherlands

**Keywords:** AF, arrhythmia surgery, atrial fibrillation, cardiovascular pathology, Cox‐Maze Procedure, surgical history

Atrial fibrillation (AF) is the most common arrhythmia and a growing health problem worldwide. The number of AF patients is increasing constantly, which can be attributed to the better detection of AF, the increase in life expectancy, and corresponding risk comorbidities predisposing to AF.^1^ The clinical relevance of AF is emphasized by a four‐ to five‐fold increasing risk for stroke, left ventricular impairment, and significantly increasing risk for morbidity and overall mortality.^1^ The original Cox‐Maze III “cut and sew” procedure, first described by Dr. James Cox et al. in 1991, was the first surgical approach to treat lone AF and its lesion set is still considered the gold standard today.^2^ Cox's idea of compartmentalizing both left and right atria, using a variety of incisions, was based on electrophysiological findings thought to be responsible for the initiation and perpetuation of AF at that time. While its effectiveness in freedom of AF is reported to be high even in the long term (92% of patients being in sinus rhythm after 14 years of which 80% were also off antiarrhythmic drugs^3^), the procedure has not been widely adopted by surgeons because of its significant degree of invasiveness, technical difficulty, and complexity. This led to a quest for less invasive alternatives. To simplify the procedure and reduce operation time, the Cox‐Maze IV, was clinically introduced in 2002.^4^ Instead of compartmentalizing the atria by cutting and sewing, a combination of bipolar radiofrequency and cryo‐energy was introduced for the creation of ablation lines, while preserving the biatrial lesion set.^5^

Another very good example of its efficacy is the present report “Midterm Outcomes of Concomitant Cox‐Maze IV: Results from a Multicenter Prospective Registry,” in which Gerdisch and colleagues describe their results from a routine performance of a standardized Cox‐Maze IV (CMIV) procedure concomitant to cardiac surgery.^6^ In total, 807 patients were analyzed from four cardiac centers over 12 years. All patients received a biatrial lesion set, as described previously,^7^ concomitant to CABG (15%), aortic valve replacement (9%), mitral valve repair or replacement (22%), or a combination of CABG/valve/multivalve (42%). Thirty‐day mortality was 3.3%, the need for pacemaker implantation was 6.3% and neurologic events were 1.3%. After 1 and 3 years, overall freedom from AF was 88.3% and 84.7%, respectively. When specifying this efficacy outcome based on preoperative AF type, freedom from AF at 1 and 3 years was 94.6% and 87.5% for paroxysmal AF, 82.1% and 81.9% for persistent AF, and 84.1% and 78.1% for longstanding persistent AF, respectively. Overall, 74% of all patients were off antiarrhythmic drugs across all study periods. Rhythm monitoring was performed by ECG (18%), 2–30 days Holter monitoring (50%), and continuous monitoring including internal loop recorders or read‐out of permanent pacemakers (32%). The authors rightfully stress that surgery was performed in complex patients, with 73% of patients undergoing at least one valve operation. As such, it is likely that the underlying mechanism of AF was secondary to valve disease and therefore different from patients with stand‐alone AF without valve disease. On the other hand, patients were known with a long AF duration with an average of 4 years and most patients (67%) were known with (longstanding)‐persistent AF. Moreover, the preoperative Euroscore was high (mean of 6.4). As such, the present study analyzed the CMIV procedure concomitant to cardiac surgery in a difficult‐to‐treat patient population that most likely developed a significant substrate for AF, even though it is possible that their valve disease initiated their AF in the first place. As such, this study demonstrates again that the CMIV procedure is a very effective and safe strategy to treat AF during several types of cardiac surgery. In a propensity analysis of matched patients undergoing a Cox‐Maze III with patients undergoing a CMIV procedure with concomitant cardiac surgery in half of the patients by Lall et al.,^8^ the CMIV is as efficacious as the Cox‐Maze III procedure with success rates of more than 90% for both procedures after 1 year, which is comparable to the outcome of the present study. Also, Ad et al.^9^ investigated the overall freedom from AF recurrence after a single CMIV procedure, but in patients undergoing stand‐alone AF surgery. After 1 and 3 years, the overall success rates were 88% and 76% off antiarrhythmic drugs, which seems comparable to the outcome of the present study. At the same time peri‐ and postoperative events including pacemaker implantation were low. This can be explained by the fact that attention was paid to the sinoatrial node when performing the superior caval vein lesion as well as ablation of the right atrial free wall.

Despite these excellent results, concomitant AF ablation during cardiac surgery was recently downgraded from a Class IA recommendation^10^ to a Class IIA recommendation in the ESC 2020 AF Guidelines.^1^ A potential explanation is the lack of strong data on improvement in quality of life (QOL) when performing add‐on arrhythmia surgery. A recent systematic review and meta‐analysis by our group illustrated that arrhythmia surgery for AF does improve QOL after both stand‐alone and concomitant arrhythmia surgery.^11^ Obviously, for patients undergoing cardiac surgery and add‐on AF surgery, the improvement in QOL can be attributed to the cardiac surgery itself as well as the additional arrhythmia surgery. However, in both groups, the gain in QOL was related to the surgical efficacy outcome (e.g., sinus rhythm after 12 months). It must be noted that this analysis contains several limitations including statistical and clinical heterogeneity due to among others the use of different lesion sets across studies and the limited sample size because not all studies reported on the SF‐36 QOL questionnaire and could therefore not be included in the meta‐analysis. Still, QOL is an important efficacy outcome and its relevance is often underestimated by physicians. Perhaps this is the reason why QOL is not widely implemented in the ESC AF Guidelines, while surgical efficacy is. The latter, often determined as the percentage of patients free from any atrial tachyarrhythmia recurrence >30 s until 12 months as defined according to the AF Guidelines,^1^ does not comprise the physical or mental improvement of the patient. Therefore, more studies should also evaluate patient‐reported outcomes including QOL to further optimize our current AF Guidelines. Maybe it is time to stop considering a recurrence lasting longer than 30 s after any AF ablation as a failure of the given therapy, but instead refer such patients for a percutaneous electrophysiological study as part of a staged hybrid ablation approach to validate the surgical ablation lines and perform endocardial touch‐up or create additional ablation lines when necessary, as this may, in turn, improve QOL even further. To facilitate this, it remains key that all patients with AF are discussed in a dedicated heart team consisting of a cardiac surgeon who is specialized in rhythm surgery and an electrophysiologist to ensure a patient‐tailored treatment approach.
